# Investigation of Pulse Waveform Index by Ambulatory Blood Pressure Monitoring and Cardiac Function∗

**DOI:** 10.1016/j.jacadv.2023.100739

**Published:** 2023-12-05

**Authors:** Bret D. Alvis, Christopher P. Siemer, Kyle M. Hocking

**Affiliations:** a Department of Anesthesiology, Vanderbilt University Medical Center, Nashville, Tennessee, USA; b Department of Biomedical Engineering, Vanderbilt University, Nashville, Tennessee, USA; c Department of Surgery, Vanderbilt University Medical Center, Nashville, Tennessee, USA

**Keywords:** waveform analysis, monitoring, heart failure

**Figure:**
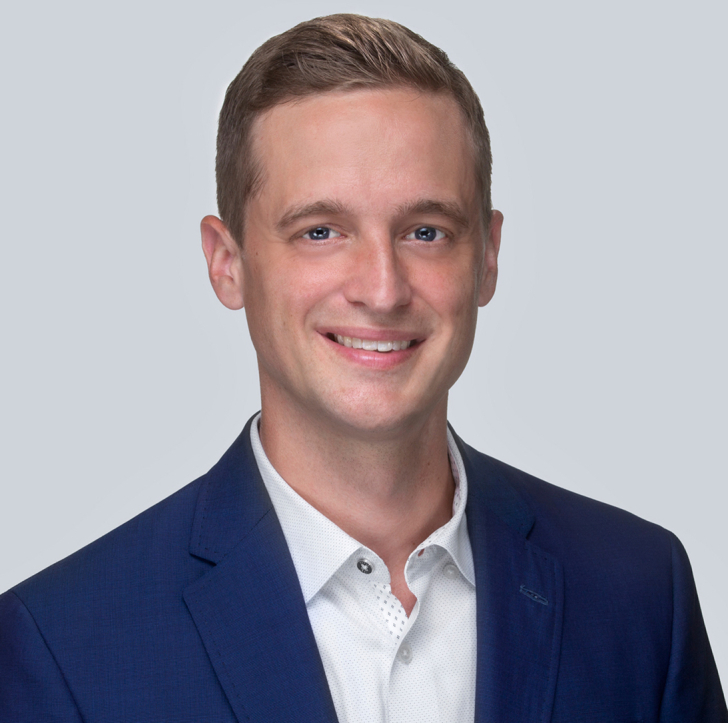

Heart failure (HF) remains a significant challenge in modern medicine, affecting millions of people worldwide.^1^ Among the key goals in HF management is the restoration of left ventricular function, which directly impacts patient outcomes and quality of life.^2^ Recent guidelines have highlighted the importance of HF with recovered ejection fraction and its correlation with improved clinical prognosis.^3^ While echocardiography, magnetic residence imaging, and catheterization are well-established methods for assessing cardiac function, their specialized, invasive nature, along with their cost, can limit their widespread use. An emerging avenue of exploration lies in the analysis of blood pressure (BP) pulse waveforms to infer cardiac function.^4^, ^5^, ^6^ This editorial delves into a recent study performed by Narita et al^7^ in this issue of *JACC: Advances*, whose team appears to have pioneered the application of an ambulatory BP monitoring device and a novel pulse waveform analysis index to evaluate cardiac systolic function in patients with HF.

This study aimed to investigate how the unique relationship between 2 key components of the BP pulse waveform (the square forward pulse wave and the amplitude measure pulse wave) changes with improved cardiac function. Although previous research has explored the link between BP pulse waveform parameters and variations in cardiac function, the authors point out that most investigations to date have involved invasive methods and/or were confined to laboratory environments that limit the practicality of a large scope of use and impact.^8^, ^9^, ^10^, ^11^, ^12^ This study describes the use of an internally developed, specialized, ambulatory cuff-oscillometric multisensor device (ABPM) in 20 patients with HF after the patients’ initial or adjusted treatments, and then again at a follow-up visit 6 to 12 months after the first reading. It provides a standard noninvasive BP reading but also enables the calculation of their described novel pulse waveform (Sf/Am) index. This index, derived from the cuff volumetric pulse waveform during ABPM, is postulated to reflect the cardiac systolic function. To investigate this, they prospectively assessed the novel index along with established echocardiographic measures in a cohort of patients with HF. In doing so, the researchers aimed to shed light on the potential utility of ABPM and its respective Sf/Am index in the evaluation of cardiac function. They hypothesized that the Sf/Am value indicates cardiac systolic function; therefore, a relationship between the Sf/Am value and left ventricular ejection fraction (LVEF) would be appreciated in the results of the prospective analysis.

The reported outcomes of their investigation do reveal a correlation between the Sf/Am index and LVEF, as well as with the change in Sf/Am and the change in LVEF. There was a statistically significant increase in the Sf/Am index from the baseline in the recovered LVEF cohort. Notably, in the nonrecovered LVEF cohort, there was no significant elevation in the Sf/Am index, gauged using ABPM, from its baseline measurement. Despite the modest sample size, consisting of only 20 subjects (11 recovered LVEF and 9 nonrecovered LVEF), the achieved statistical significance carries some weight. These data support the hypothesis that the Sf/Am index is related to cardiac systolic function. This observation is the first to describe this relationship and the prospect of a noteworthy connection between the 2 in each population, where the presence of a substantial effect is evident.

This observation underscores the potential of the Sf/Am index as a surrogate marker of cardiac recovery in patients with HF. Moreover, this study's exploration of changes in ambulatory BP profiles provides a comprehensive perspective on the hemodynamic shifts that accompany cardiac function improvement. By analyzing 24-hour, daytime, and nighttime parameters, this research adds depth to our understanding of how these indices correlate with cardiac changes throughout the day with and without LVEF recovery. This study's reliance on ABPM, a well-established method for obtaining accurate and reliable BP readings, enhances its clinical relevance. ABPM not only overcomes the limitations of sporadic office-based measurements but also captures the dynamic nature of BP, thereby offering a more comprehensive picture of patients' conditions. The calculated Sf/Am index, derived from the ABPM data, provides a potential that could provide valuable insight into a HF patient’s dynamic, real-time cardiac condition.

This study has certain limitations that warrant consideration. The sample size, which is suitable for preliminary exploration, is the largest limitation, and very little can be truly concluded based on this analysis. We agree with the authors’ conclusion that a large-sample observational study is necessary to establish the usefulness of this Sf/Am index in patients with HF using ABPM. Furthermore, the study design lends itself to certain biases such as the enrollment of patients during hospitalization or outpatient visits, which could impact the generalizability of the findings. In the future, this study will open exciting avenues for further investigation. Prospective research could delve into longitudinal changes in the Sf/Am index and their correlation with cardiac function over various time/treatment periods. Additionally, a broader patient cohort may allow for the identification of subgroups that benefit from this assessment approach. Incorporating more diverse patient populations, including those with different HF etiologies and comorbidities, could yield valuable insights into the applicability of the Sf/Am index across the HF spectrum.

Beyond the clinical realm, the potential integration of wearable technology offers exciting prospects. The advent of smartwatches and other wearable devices has increased the accessibility of continuous health monitoring. Integrating the Sf/Am index into such platforms could empower patients to play a more proactive role in cardiac health management.

Narita et al bridged the gap between traditional cardiac assessment methods and the innovative world of ambulatory monitoring and pulse waveform analysis. By introducing the Sf/Am index as a potential surrogate marker for cardiac systolic function, this study expands the possibilities for HF management strategies. The findings of this study emphasize the value of ABPM in providing a comprehensive view of cardiovascular dynamics and its potential to predict cardiac recovery. While acknowledging its limitations, this study paves the way for future research endeavors that could reshape the landscape of HF evaluations.

## Funding support and author disclosures

The authors have reported that they have no relationships relevant to the contents of this paper to disclose.
